# Gallbladder Squamous Cell Carcinoma with Diffuse β-hCG Expression: A Case Report and Review of the Literature

**DOI:** 10.1007/s12029-025-01308-7

**Published:** 2025-09-09

**Authors:** Shengliang He, Dustin E. Bosch, Alan Hemming

**Affiliations:** 1 Department of Surgery, Division of Transplant & Hepatobiliary Surgery, Organ Transplant Center, University of Iowa Health Care Medical Center, 200 Hawkins Dr, Iowa City, IA USA; 2 Department of Pathology, Roy J. and Lucille A. Carver College of Medicine, University of Iowa, Iowa City, IA USA; 3 Holden Comprehensive Cancer Center, Iowa City, IA USA

**Keywords:** Gallbladder squamous cell carcinoma, β-human chorionic gonadotropin, Tumor biology, Biomarker

## Abstract

**Purpose:**

Gallbladder squamous cell carcinoma (SCC) is a rare subtype of gallbladder malignancy, comprising only 1–4% of cases. Ectopic expression of β-human chorionic gonadotropin (β-hCG) has been described in various epithelial cancers and is associated with aggressive behavior. We report the first known case of gallbladder SCC with diffuse β-hCG expression and markedly elevated serum β-hCG levels, aiming to explore its clinicopathological implications and potential as a prognostic biomarker.

**Methods:**

We present the clinical, radiologic, surgical, and pathological findings of a patient with a large gallbladder mass. A literature review was conducted to contextualize our findings.

**Results:**

The patient underwent open cholecystectomy with hepatic segment IVb/V resection. Final pathology revealed poorly differentiated SCC with acantholytic features and diffuse β-hCG immunoreactivity. Two metastatic lymph nodes were identified in the low hepatoduodenal nodal basin. Postoperative serum β-hCG was significantly elevated. Despite surgical resection, the patient experienced rapid disease recurrence and progression.

**Conclusion:**

This case represents the first documented instance of β-hCG-expressing gallbladder SCC. Diffuse β-hCG expression may reflect a dedifferentiated and aggressive tumor phenotype associated with early metastasis and poor prognosis. Further studies are warranted to investigate the prognostic and biological significance of β-hCG in gallbladder SCC and its potential role as a biomarker.

## Background

Gallbladder cancer is the most common malignancy of the biliary tract, accounting for approximately 80–90% of all biliary tract cancers worldwide [1, 2]. Histologically, about 90% of gallbladder cancers are adenocarcinomas and about 5% have mixed adenosquamous differentiation [3]. In contrast, pure squamous cell carcinoma (SCC) of the gallbladder is rare and typically associated with more advanced disease at presentation, delayed diagnosis, and a poorer prognosis [4–6]. We report a case of a patient presenting with a large gallbladder mass, histopathologically confirmed as squamous cell carcinoma, demonstrating diffuse β human chorionic gonadotropin (hCG) expression—a finding that, to our best knowledge, has not been previously reported in the literature.

## Case Presentation

**Fig. 1:**
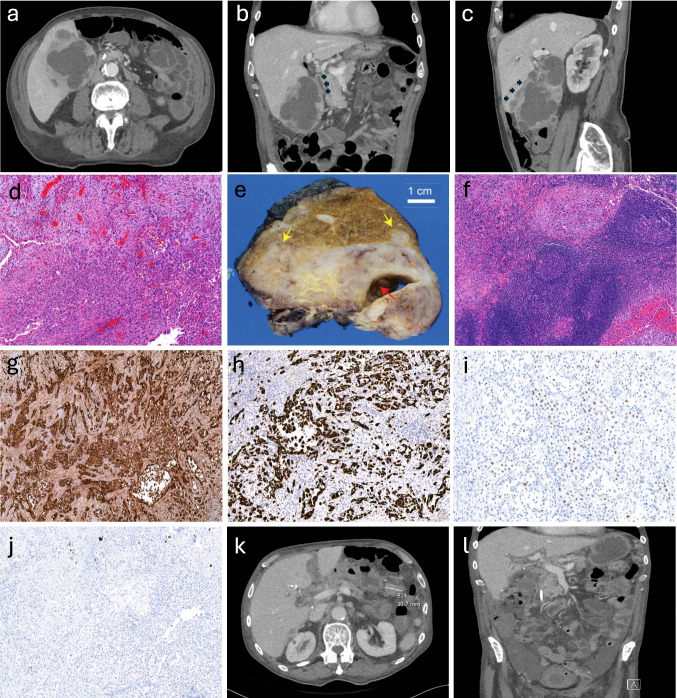
**a**-**l**. Representative images and pathology of a b-hCG positive acantholytic squamous cell carcinoma. **a**, preop CT axial view, large heterogenous gallbladder mass; **b**, preop CT coronal view, large gallbladder mass compressing the duodenum (black arrows); **c**, preop CT sagittal view, gallbladder mass direct invading the adjacent liver parenchyma (black arrows); **d**, H&E of the primary tumor with poorly differentiated carcinoma cells and acantholytic features (5X); **e**, gross photograph of the sectioned mass after fixation showing the mass with relationship to the gallbladder lumen (red arrow) and liver bed invasion (yellow arrows); **f**, metastatic carcinoma in a station 13 lymph node (5X); **g**, diffusely positive b-hCG immunohistochemistry (5X); **h**, diffusely positive CK5/6 (5X); **i**, variably positive p40 (10X); **j**, negative SALL4 (5X); **k**, mesenteric nodule, postop CT; **l**, pelvic tumor deposit, ascites with thickened bladder wall. CT, computed tomography

An 82-year-old male initially presented to an outside hospital with a three-month history of fatigue, right upper quadrant abdominal pain, and unintentional weight loss. He was noted to have a fever and leukocytosis with a white blood cell count of 23,000/μL. Computed tomography (CT) imaging revealed an enlarged gallbladder with heterogeneous components, involvement of hepatic segments IVb and V, mass effect on the second portion of the duodenum, and compression of the extrahepatic bile duct with associated intrahepatic biliary ductal dilatation (Fig. 1a-c).

The patient was started on broad-spectrum antibiotics, and endoscopic retrograde cholangiopancreatography (ERCP) was performed with placement of a biliary stent. Brush cytology obtained during ERCP was negative for malignancy. Additionally, upper gastrointestinal endoscopy was performed, revealing no pathological abnormalities. Endoscopic ultrasound (EUS)-guided biopsy of the gallbladder mass demonstrated isolated malignant cells with prominent nucleoli and irregular nuclear membranes, diagnostic of carcinoma. A concurrently biopsied enlarged celiac lymph node revealed reactive changes without evidence of malignancy. Following biliary stent placement, the patient had a mildly elevated alkaline phosphatase level (149 U/L), while the remainder of the LFT remained within normal limits. Despite a week of antibiotic therapy, the patient showed no clinical improvement and was transferred to our center for further evaluation and management.

The working diagnosis was gallbladder cancer with superimposed infection. Further staging with CT imaging revealed no evidence of distant metastasis or additional nodal involvement. Given the failure of antibiotic therapy and concerns regarding potential tumor seeding with percutaneous drainage, cholecystostomy tube was deemed inappropriate. We elected to proceed with surgical resection. The patient underwent open cholecystectomy with en-bloc resection of hepatic segments IVb and V. The gallbladder was markedly enlarged and rock-hard on palpation. There appeared to be extension of either tumor or associated inflammation into the adjacent hepatic parenchyma. The omentum, which was densely adherent to the gallbladder surface, was resected en-bloc with the gallbladder specimen. The cystic duct margin was negative for tumor involvement, and the common bile duct appeared grossly uninvolved. He tolerated the procedure well and was discharged on postoperative day five without complications.

Final pathology revealed a poorly differentiated SCC with acantholytic features (Fig. 1d), involving the liver parenchymal margin (Fig. 1e). There was complete diagnostic consensus among three gastrointestinal pathologists who reviewed the case. Coexistent cholelithiasis was also noted. Lymph node involvement was noted at station 13 (2/2, Fig. 1f), while nodes at stations 8 (0/3) and 12 (0/2) were negative. While margins were grossly negative, microscopic examination showed microscopic involvement at the hepatic resection margin. The tumor exhibited infiltrative architecture, marked cytologic atypia, and abundant eosinophilic cytoplasm (Fig. 1d). Immunohistochemically, the neoplastic cells were diffusely positive for pancytokeratin, CK7, CK5/6, CK903, and β-hCG, with variable positivity for p40 (Fig. 1g-i). The tumor was negative for a broad range of markers including CDH17, CK20, SALL4, ERG, Oct4, PLAP, CD30, arginase, glypican-3, and HepPar1. A mucicarmine stain was negative. Expression of SMAD4, BRG1, and BAP1 was retained. While the tumor’s acantholytic features represent an uncommon histologic pattern that can mimic gland-forming adenocarcinoma, its overall morphology and immunoprofile were consistent with a pure squamous cell carcinoma, lacking true glandular or mucinous differentiation. Thorough sampling revealed no distinct gland-forming components, excluding adenosquamous carcinoma. Since the carcinoma showed high-level expression of β-hCG (Fig. 1g), a germ cell tumor was further excluded with a panel of immunohistochemistry, including SALL4 (Fig. 1j).

Of note, the patient’s preoperative carcinoembryonic antigen (CEA) level was within normal limits, while carbohydrate antigen (CA) 19–9 was mildly elevated at 51 U/mL (reference range: < 34 U/mL). Following the discovery of diffuse β-hCG expression in the tumor specimen, post operative serum β-hCG was obtained and found to be markedly elevated at 168 mIU/mL (reference range for males: < 2 mIU/mL). However, no preoperative β-hCG measurement was available for comparison.

A repeat CT scan at six weeks postoperatively revealed multiple new mesenteric and omental deposits, highly suspicious for peritoneal carcinomatosis (Fig. 1k-l). The patient was evaluated by the hematology/oncology team, and adjuvant therapy had been planned. However, due to rapid disease recurrence, he elected for comfort-focused care and unfortunately passed away on postoperative day 46 before treatment could be initiated.

## Discussion

Squamous differentiation of gallbladders cancer is uncommon, observed in approximately 12% of gallbladder malignancies overall, yet “pure” SCC constitutes only 1–4% of all cases [6–8]. Its pathogenesis remains poorly understood. Proposed mechanisms include squamous metaplasia within a pre‑existing adenocarcinoma, malignant transformation of heterotopic squamous epithelium, and malignant transformation of metaplastic squamous epithelium [5, 9]. Notably, minute or “pure” squamous cell carcinomas lacking an adenocarcinoma component or adjacent metaplastic/heterotopic squamous epithelium have been described, suggesting a distinct tumorigenic pathway [10].

Takahashi et al. recently conducted a systematic review of gallbladder SCC, demonstrating that, similar to gallbladder adenocarcinoma, SCC exhibits a predilection for females and elderly populations, with a median age at diagnosis of 65–70 years [5]. While the clinical presentation of gallbladder SCC closely mirrors that of other gallbladder malignancies, patients with SCC typically present with larger tumor dimensions and more advanced pathological stages upon initial diagnosis [5–7]. Radiographically, gallbladder SCC often manifests as a gallbladder mass, with or without direct hepatic or adjacent soft tissue invasion. Notably, up to 80% of cases are associated with concurrent cholelithiasis [11]. The diagnosis of gallbladder SCC is primarily established through histopathological examination. Histologically,"pure"SCC is defined by exclusive squamous differentiation, frequently demonstrating prominent keratinization and intercellular bridges, with no evidence of malignant glandular components [12]. In cases where both squamous and glandular differentiation coexist, the tumor is classified as adenosquamous carcinoma. Although no definitive threshold for squamous component percentage has been established, current diagnostic criteria typically require squamous differentiation to constitute at least 25–30% of tumor volume [13, 14]. The optimal therapeutic approach involves radical surgical resection combined with systemic chemotherapy, which appears to confer the most favorable outcomes. Nevertheless, the overall prognosis remains markedly inferior to that of gallbladder adenocarcinoma, likely attributable to the tumor’s aggressive biological behavior [4, 9]. A recent analysis of 1084 cases from the national cancer database revealed a significant disparity in median overall survival between gallbladder SCC and adenocarcinoma (9 months vs. 17 months, respectively; *p* < 0.001). This survival difference persisted even among patients who underwent R0 resection (13 months vs. 29 months, respectively; *p* < 0.001) [15].

Serum tumor markers have been extensively studied for their potential roles in diagnosis, disease monitoring, and treatment response in various cancers; however, their utility in gallbladder cancer has yielded unsatisfactory and inconsistent results [16, 17]. Traditional tumor markers such as serum CEA, CA 19–9, CA 125 and CA 242 have been associated with gallbladder cancer, though their reported sensitivity and specificity vary across studies [18–20]. These markers are not specific to gallbladder cancer. CA 19–9, for example, can also be elevated in patients with other malignancies or benign causes of jaundice. Some studies have suggested that combining multiple markers may improve diagnostic sensitivity [21]. Most of the guidelines, like National Comprehensive Cancer Network guideline, recommend to consider obtaining CEA or CA 19–9 as baseline test in gallbladder mass workup [22]. Emerging evidence suggests that liquid biopsy, such as circulating tumor DNA, microRNAs, and circulating tumor cells, hold promise for the diagnosis and monitoring of gallbladder cancer. However, despite encouraging preliminary findings, further validation is required before these modalities can be incorporated into routine clinical practice [17, 23].

Unsurprisingly, most of the published literature on gallbladder malignancies has focused on adenocarcinoma, with limited data available specifically on gallbladder SCC. The association between tumor markers such as CA 19–9 or CEA and gallbladder SCC remains poorly defined. SCC originating from non-glandular sites, such as the head and neck or skin, typically do not secrete these markers [7]. This discrepancy may be attributable to differences in cellular origin; while squamous cell carcinomas of the head, neck, or skin arise from native squamous epithelium, gallbladder SCC is thought to originate from biliary epithelium that has undergone squamous metaplasia.

hCG plays diverse physiological and pathological roles and is widely utilized as a tumor biomarker in selected malignancies [24]. The term “chorionic gonadotropin” derives from “chorion,” referencing the extra-embryonic membrane, and “gonadotropin,” indicating its function as a hormone that stimulates the gonads, particularly in promoting ovarian steroidogenesis. Beyond its physiological role, hCG is expressed by both trophoblastic and non-trophoblastic tumors and has been implicated in several hallmarks of cancer biology, including cellular transformation, angiogenesis, metastatic potential, and immune evasion [25]. Elevated hCG concentrations have been observed in tumors not of trophoblastic origin, particularly carcinomas of the pancreas, ovary, and breast, suggesting ectopic hormone production by either the entire tumor population or, more plausibly, a subclone of poorly differentiated malignant cells [26]. The presence of ectopic hCG expression in these tumors has prompted speculation that the genomic elements responsible for hCG synthesis may have phylogenetically ancient origins [27].

Furthermore, tumor-derived production of hCG is often associated with the release of free hormone subunits, particularly the beta subunit (β-hCG), into the circulation. This has important clinical implications for cancer monitoring. β-hCG is routinely utilized as a biomarker for the diagnosis, staging, and surveillance of germ cell tumors, including testicular and ovarian malignancies, as well as gestational trophoblastic disease. Ectopic β-hCG secretion has also been documented in various epithelial malignancies, including those of the lung, ovary, stomach, breast, and bladder [28]. However, this phenomenon is likely underrecognized, as β-hCG is not routinely measured during the diagnostic workup of most non-germ cell cancers in adults.

Studies have demonstrated that approximately 30% of patients with epithelial malignancies exhibit elevated serum β-hCG levels, and up to 40% of carcinomas stain positive for β-hCG by immunohistochemistry [25]. While the incidence is too low to support its use as a screening tool, β-hCG expression has been strongly correlated with higher tumor grade, advanced stage, and decreased overall survival [28, 29]. Structural homology and in vitro studies indicate that β-hCG may achieve this by interfering with the transforming growth factor-beta receptor complex, a mechanism that could help explain its link with poor prognosis and rapid tumor progression [24]. A comprehensive literature review was conducted to identify previously reported cases and studies relevant to gallbladder SCC, β-hCG expression in non-trophoblastic tumors, and potential biomarkers in biliary tract malignancies. Searches were performed using PubMed, Embase, and Scopus databases, utilizing combinations of the following keywords and MeSH terms: “gallbladder carcinoma,” “squamous cell carcinoma,” “gallbladder cancer,” “β-human chorionic gonadotropin,” “β-hCG,” “tumor markers,” “ectopic hormone expression,” and “biomarkers in biliary tract cancer.” Search filters were applied to include articles in English, studies involving human subjects, and peer-reviewed publications. Reference lists of selected articles were also reviewed to identify additional relevant reports and case studies not captured in the initial database query. To the best of our knowledge, β-hCG expression has been reported in gallbladder adenocarcinoma, undifferentiated carcinoma, and choriocarcinoma, but not in pure squamous cell carcinoma [30–38]. Reported cases typically involve middle-aged female patients presenting at an advanced stage, and despite receiving active treatment, the prognosis remains poor. A summary of reported patient characteristics in primary gallbladder cancers exhibiting β-hCG expression is presented in Table 1.

**Table 1:** Characteristics of primary gallbladder cancer with beta human chorionic gonadotropin expression from reported cases

Published Year	Author	Numbers of patients	Age	Gender	Synchronous liver metastasis	Histology type	Treatment	Outcome
2023	Wang et al	1	58	Female	Yes	Adenocarcinoma	Surgery, chemo and ICI	No recurrence at one year
2018	Leostic et al	1	31	Female	No	Adenocarcinoma with an undifferentiated component	Surgery	Metachronous liver metastasis weeks after surgery
2014	Gato et al	1	61	Female	Yes	Adenocarcinoma	Chemo	NA
2010	Sato et al	1	79	Male	NA	Adenocarcinoma	Surgery	No recurrence at 11 months
2008	Macdonald et al	1	41	Female	Yes	Trophoblast tumor	Chemo	Death at 7 months
2001	Wang et al	1	48	Female	No	Adenocarcinoma and choriocarcinoma	Surgery, chemo	Metachronous liver metastasis. Death at one year
1991	Abu-Farsakh et al	1	29	Female	NA	Adenocarcinoma and choriocarcinoma	NA	Death at one month
1990	Fukuda et al	1	83	Female	Yes	Adenosquamous cell carcinoma	NA	Death
1988	Guo et al	9	65, 61–69 (mean, range)	66.7% Female	NA	Undifferentiated carcinoma with pleomorphic cell type (44.4%, 4/9) or spindle cell/pseudosarcomatous type (55.6%, 5/9)	55.6% Surgery, 44.4% chemo	44.4% (4/9) death within one year, 11.1% (1/9) alive at 19 months, 44.4% (4/9) NA

Our patient final pathology was pure SCC of the gallbladder at an advanced stage (pT3N1, AJCC 8th edition), a rare and aggressive subtype characterized by locally invasive growth. The tumor directly invaded adjacent hepatic parenchyma and exhibited lymphatic spread and metastases to low hepatoduodenal nodes (position 13), while sparing the higher portal nodes (position 12) and common hepatic artery (position 8) nodal basins. Histopathological evaluation revealed diffuse β-hCG immunoreactivity, with corresponding markedly elevated postoperative serum β-hCG levels, consistent with an aggressive tumor phenotype. Regrettably, preoperative baseline or serial serum β-hCG measurements were unavailable for comparative analysis in this case. While comprehensive metastatic workup yielded negative results, the possibility of an occult malignancy with ectopic β-hCG production cannot be entirely excluded as the source of the observed β-hCG elevation. The elevated preoperative CA 19–9 level may reflect biliary manipulation or mild compression of the bile duct by the mass. Despite aggressive surgical resection, the patient experienced early recurrence with disseminated disease.

## Conclusion

This case represents the first reported instance of gallbladder squamous cell carcinoma with diffuse β-hCG expression. The tumor demonstrated aggressive biological behavior, including direct hepatic invasion, skip metastases to distant lymph nodes and early recurrence. The expression of β-hCG in this context may represent a novel biomarker with potential implications for tumor classification, prognostication, and therapeutic stratification. Proteomic profiling and further investigation into the role of β-hCG in gallbladder SCC are warranted.

## Data Availability

No datasets were generated or analysed during the current study.

## Abbreviations

CA: Carbohydrate antigen
CEA: Carcinoembryonic antigen
CT: Computed tomography
ERCP: Endoscopic retrograde cholangiopancreatography
EUS: Endoscopic ultrasound
hCG: Human chorionic gonadotropin
SCC: Squamous cell carcinoma
